# The Correlation Between Bronchopulmonary Dysplasia and Platelet Metabolism in Preterm Infants

**DOI:** 10.3389/fped.2021.670469

**Published:** 2021-11-24

**Authors:** Longli Yan, Zhuxiao Ren, Jianlan Wang, Xin Xia, Liling Yang, Jiayu Miao, Fang Xu, Weiwei Gao, Jie Yang

**Affiliations:** Department of Neonatology, Guangdong Women and Children Hospital, Guangzhou Medical University, Guangzhou, China

**Keywords:** bronchopulmonary dysplasia, platelet, TPO, megakaryocyte, CD62P, CD63

## Abstract

**Background:** Platelets play an important role in the formation of pulmonary blood vessels, and thrombocytopenia is common in patients with pulmonary diseases. However, a few studies have reported on the role of platelets in bronchopulmonary dysplasia.

**Objective:** The objective of the study was to explore the relationship between platelet metabolism and bronchopulmonary dysplasia in premature infants.

**Methods:** A prospective case-control study was performed in a cohort of premature infants (born with a gestational age <32 weeks and a birth weight <1,500 g) from June 1, 2017 to June 1, 2018. Subjects were stratified into two groups according to the diagnostic of bronchopulmonary dysplasia: with bronchopulmonary dysplasia (BPD group) and without bronchopulmonary dysplasia (control group). Platelet count, circulating megakaryocyte count (MK), platelet-activating markers (CD62P and CD63), and thrombopoietin (TPO) were recorded and compared in two groups 28 days after birth; then serial thrombopoietin levels and concomitant platelet counts were measured in infants with BPD.

**Results:** A total of 252 premature infants were included in this study. Forty-eight premature infants developed BPD, 48 premature infants without BPD in the control group who were matched against the study infants for gestational age, birth weight, and admission diagnosis at the age of postnatal day 28. Compared with the controls, infants with BPD had significantly lower peripheral platelet count [BPD vs. controls: 180.3 (24.2) × 10^9^/L vs. 345.6 (28.5) × 10^9^/L, *p* = 0.001]. Circulating MK count in the BPD group was significantly more abundant than that in the control group [BPD vs. controls: 30.7 (4.5)/ml vs. 13.3 (2.6)/ml, *p* = 0.025]. The level of CD62p, CD63, and TPO in BPD group was significantly higher than the control group [29.7 (3.1%) vs. 14.5 (2.5%), 15.4 (2.0%) vs. 5.8 (1.7%), 301.4 (25.9) pg/ml vs. 120.4 (14.2) pg/ml, all *p* < 0.05]. Furthermore, the concentration of TPO was negatively correlated with platelet count in BPD group with thrombocytopenia.

**Conclusions:** Our findings suggest that platelet metabolism is involved in the development of BPD in preterm infants. The possible mechanism might be through increased platelet activation and promoted TPO production by feedback.

## Introduction

Bronchopulmonary dysplasia (BPD) is one of the common complications in neonatal intensive care units (NICU) and is considered to be the main cause of death in preterm infants (1). In recent years, with advances in perinatal and neonatal care of preterm infants such as antenatal steroid usage, surfactant therapy, and ventilation strategies, the survival rate of premature infants also increases, resulting in an increasing incidence of BPD. BPD severely influences premature infants by increasing their risk of respiratory infection, asthma, and chronic obstructive pulmonary distress in their later life (2–4).

Currently, alveolar and microvascular arrest is considered as the pathological feature of clinical bronchopulmonary dysplasia (5), the development of alveolar microvascular can promote the formation of alveoli structure; therefore, the role of pulmonary vascular development, especially in pulmonary microvascular development in BPD is getting more and more attention.

The platelet has been recognized as a multifunctional cell, besides hemostasis and thrombosis. The platelet plays an essential role in the formation and development of pulmonary blood vessels, as demonstrated by numerous studies (6, 7). Some clinical retrospective studies (8, 9) found that mean platelet volume might predict the occurrence of BPD, and also, a study (10) has shown that higher platelet count is an independent factor of the development of moderate–severe BPD, and the research (11) found that a higher platelet transfusion threshold can increase the risk of BPD in premature infants. So, we speculate that platelets are involved in the occurrence of BPD, and a few studies have been reported in this regard.

In the present study, we evaluated this hypothesis by comparing peripheral platelet count, circulating MK count, platelet-activating markers, (CD62P and CD63) 28 days after birth in two groups, and the relationship between TPO expression and platelet count in infants with BPD.

## Materials and Methods

### Study Design and Population

This is a prospective study performed at the Neonatal Intensive Care Unit (NICU), Guangdong Women and Children Hospital from June 1, 2017 to June 1, 2018. This study was approved by the ethics committee of Guangdong Women and Children Hospital. Preterm infants cared for in our center were enrolled if they satisfied the following recruitment criteria: (1) gestation <32 weeks, (2) birth weight <1.5 kg, (3) requiring mechanical ventilation for the treatment of respiratory distress syndrome for at least 3 days during the first weeks of life, (4) ventilator and/or oxygen dependent at the time of enrollment, and (5) presence of clinical and radiologic signs of BPD.

Prospectively determined exclusion criteria included the following conditions: (1) congenital abnormalities; (2) infection (bacterial infection confirmed by positive blood culture or viral infection confirmed by serological test or viral culture); (3) evidence of complications of perinatal asphyxia including an Apgar score <3 at 1 or 5 min after birth, evidence of hypoxic–ischemic encephalopathy, acute tubular necrosis, or transient myocardial ischemia; and (4) identifiable hematologic disease.

The infants who were matched against the study infant for gestational age, birth weight, and admission diagnosis were recruited as control group at their age of postnatal day 28.

### Definition of Clinical Variables

The diagnosis of BPD in preterm birth was assessed using the consensus definition of the National Institute of Child Health and Human Development (NICHD). Briefly, BPD was defined as the need for supplemental oxygen for more than 28 days and the severity was assessed according to the oxygen concentration required at 36 weeks PMA or discharge (12). Neonatal thrombocytopenia was defined arbitrarily as a platelet count of <150,000 mm^3^. Neonatal respiratory distress syndrome (NRDS) was defined according to Gomella's Neonatology (13).

### Data Collection

The following data were retrieved from the electronic medical record, including maternal disease, gestation, birth weight, gender, Apgar score, NRDS, ventilation mode, blood testing (hemoglobin, white cell count, platelet count, blood gas) using the samples collected within 1 h after birth from the peripheral arterial of the infants.

### Measurements

#### Blood Sampling

In enrolled premature infants at their age of postnatal day 28, during blood sampling for routine laboratory investigation, 1 ml of blood was drawn from the peripheral arterial and placed into an EDTA tube. After mixing gently, 40 μl of whole blood was used as an aliquot for the detection of CD62P and CD63 expressions by flow cytometry. After centrifugation at 400 × *g*, plasma was separated from the blood and preserved at −80°C for TPO level detection. The cell pellet was resuspended in phosphate-buffered saline (PBS) for circulating MK isolation. All samples were processed within 1 h of collection.

#### Platelet Counts and Circulating Megakaryocyte Counts

Blood platelets were quantified by a Japanese Sysmex KX221 automatic blood cell analyzer. Circulating MK count was estimated as previously described (14). Briefly, samples were passed through a syringe filter holder (Millipore, Ireland) containing a polycarbonate membrane with an aperture of 5 μm at 37°C and then washed with 2 ml of saline. After the removal of the membrane, it was dried quickly and slightly with a heater and was left overnight at room temperature.

#### CD62P and CD63 Expressions

CD62P and CD63 were estimated by flow cytometry (FACSCalibur, Becton-Dickinson, San Jose, CA, USA) with the procedure details as previously described (14). In short, 40 μl of whole blood was taken at room temperature and stained with the monoclonal antibody at a saturated concentration for 30 min in dark. FITC-conjugated CD61 is regarded as a platelet-specific monoclonal antibody, and anti-cd62p and anti-cd63 combined with PE in dichroism analysis were used as platelet activation markers. After incubation, 1 ml of PBS containing 1% paraformaldehyde was added to each tube. The samples were fixed at 4°C for 24 h for flow cytometry detection. CD62P and CD63 were detected in 20,000 platelets. All antibodies were purchased from Abcam.

#### Plasma Thrombopoietin Concentration

TPO levels were measured using a commercially available ELISA (R&D Systems Quantikine Human TPO ELISA kit, Minneapolis, MN, USA), with a lower limit of detection of 15 pg/ml.

### Statistical Analysis

Statistical analyses between the two groups were performed using two-tailed Student's *t*-test. One-way analysis of variance was performed to compare the relationship between TPO concentration and platelet count at each time point in BPD group. A value of *p* < 0.05 was considered statistically significant. All values were expressed as mean (SEM) unless otherwise stated. All statistical analyses were done using SPSS 20.0.

## Results

### Demography Characteristics

**Figure 1:**
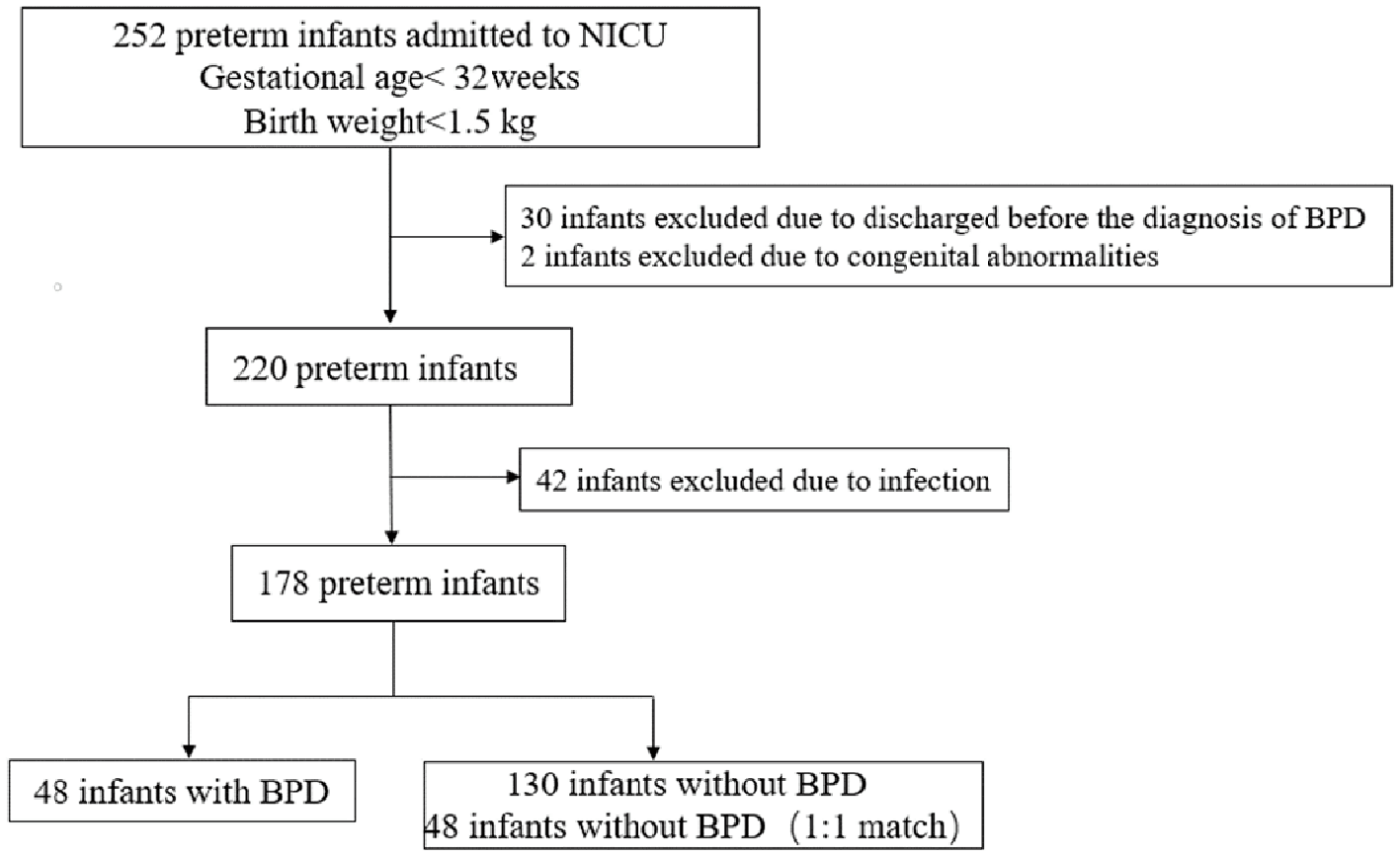
Flowchart of case selection and analysis.

A total of 252 eligible premature infants were admitted to our NICU during the study period, after applying exclusion criteria, 178 premature infants were included, in which 48 premature infants diagnosed with BPD, 134 premature infants without BPD, and 48 premature infants in the control group were matched 1:1 according to gestational age, birth weight, and admission diagnosis at their age of postnatal day 28 (Figure 1).

The demographic characteristics of the study infants are shown in Table 1.

**Table 1:** Demographic characteristics of infants with BPD.

**Variable**	**BPD**	**Control**	***p*-Value**
	**(*n* = 48)**	**(*n* = 48)**	
Gestation (weeks)	28.5 (0.5)	29.7 (0.43)	0.17
Birth weight (g)	1,040 (60)	1,220 (60)	0.14
**Gender**			
Female	27	24	0.23
Male	21	14	0.25
**Maternal disease**			
PIH	20	16	0.32
IUGR	4	2	0.25
PROM	6	4	0.30
Oligohydramnios	3	1	0.22
Twin–twin transfusion	1	1	0.5
APH	3	4	0.36
Nil	4	2	0.42
**Apgar score**			
At 1 min	7	7	0.55
At 5 min	8	9	0.45
**Admission diagnosis**			
Prematurity	48	48	
RDS	48	44	
Hemoglobin (g/ml)	11.0 (0.3)	10.4 (0.4)	0.18
White cell count (x10^5^/L)	10.5 (0.3)	11.8 (1.5)	0.55
Platelet count (x10^9^/L)	170.1 (15.4)	343.1 (23.4)	<0.001
**Ventilation duration (days)**			
IPPV	20 (0.8)	9 (0.4)	<0.001
CPAP	29 (1.7)	15 (0.7)	<0.001
HFOV	12 (1.3)	7 (0.4)	0.08
**Blood gas**			
PH	7.37 (0.04)	7.39 (0.03)	0.25
PaO_2_	6.31 (0.4)	5.7 (0.4)	0.34
PaCO_2_	5.0 (0.4)	5.1 (0.3)	0.83
FiO_2_	0.27 (0.03)	0.21 (0.3)	<0.001

*PIH, pregnancy-induced hypertension; IUGR, intra-uterine growth retardation; PROM, prolonged rupture of membrane; SLE, systemic lupus erythematosus; IPPV, intermittent positive pressure ventilation; CPAP, continuous positive airway pressure; HFOV, high-frequency oscillatory ventilation; BPD, bronchopulmonary dysplasia*.

We studied 48 infants in the BPD group (female: 27; male: 21) and 48 infants in the control group (female: 24; male: 24). The infants in the BPD group and control group were similar in their demographic characteristics including gestational age and birth weight. However, the infants with BPD had been exposed to a significantly higher FiO_2_ and had a significantly longer ventilation duration (Table 1). Among the infants with BPD, 30 infants had a peripheral platelet count <150 × 10^9^/L, while none of the infants in the control group had a platelet count <150 × 10^9^/L. Infants in both groups received similar conventional treatment according to the unit protocol.

Thirty thrombocytopenic infants in the BPD group were studied serially at postnatal age of day 28, day 35, and day 42, respectively. The platelet count in all these infants returned to normal by the age of day 42.

### Peripheral Platelet Count

**Figure 2:**
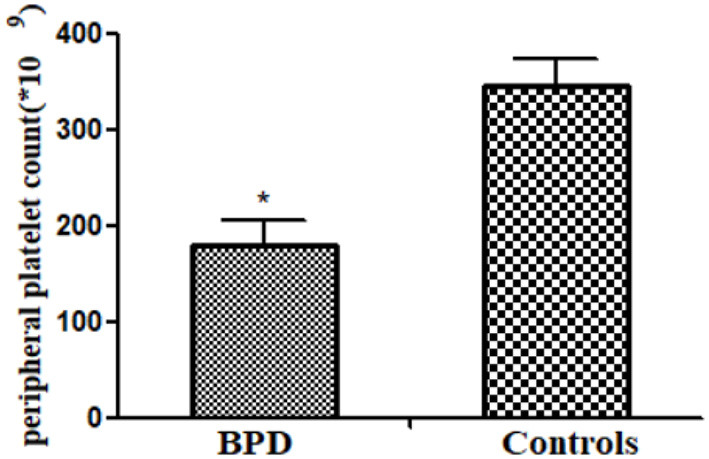
Peripheral platelet counts in infants of bronchopulmonary dysplasia (BPD) and control groups (*p* = 0.001). *p < 0.05.

Peripheral platelet count in the BPD group was significantly lower than that in the control group [BPD vs. controls: 180.3 (24.2) × 10^9^/L vs. 345.6 (28.5) × 10^9^/L, *p* = 0.001], as shown in Figure 2.

### Circulating Megakaryocyte Count

**Figure 3:**
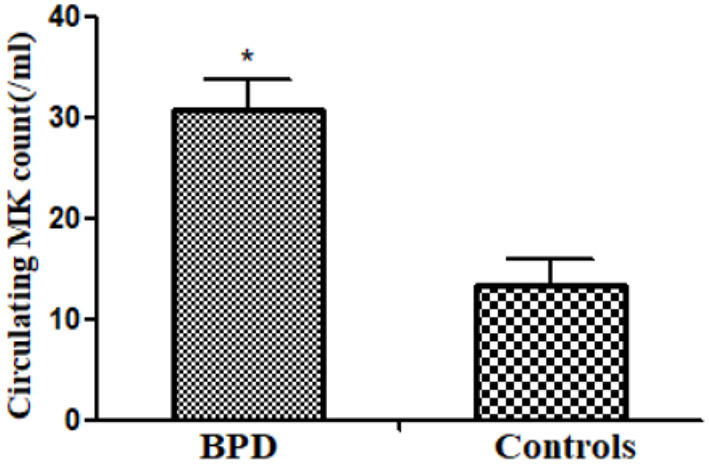
Peripheral megakaryocyte (MK) counts in infants of BPD and control groups (*p* = 0.025). *p < 0.05.

Circulating MK count in the BPD group was significantly more abundant than that in the control group [BPD vs. controls: 30.7 (4.5)/ml vs. 13.3 (2.6)/ml, *p* = 0.025], as shown in Figure 3.

### CD62P and CD63 Expressions on Platelets

**Figure 4:**
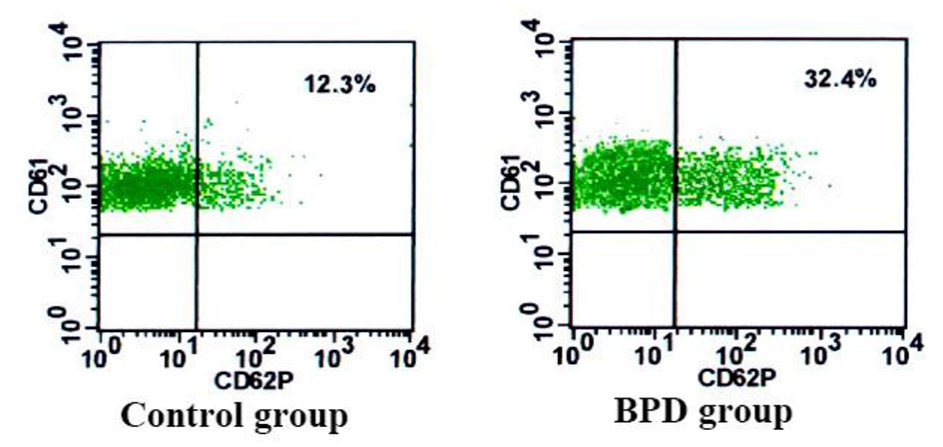
The percentage of CD61+CD62P+ in the control group and BPD group.

**Figure 5:**
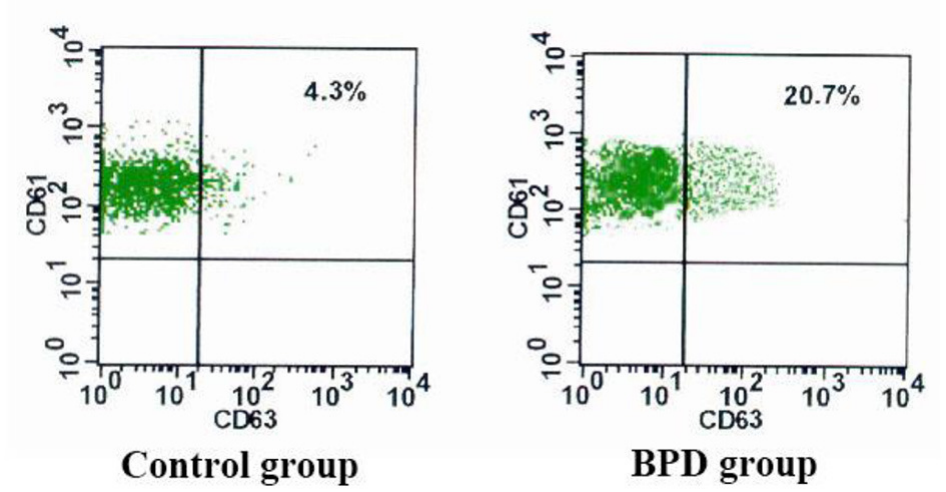
The percentage of CD61 + CD63 in the control group and BPD group.

**Figure 6:**
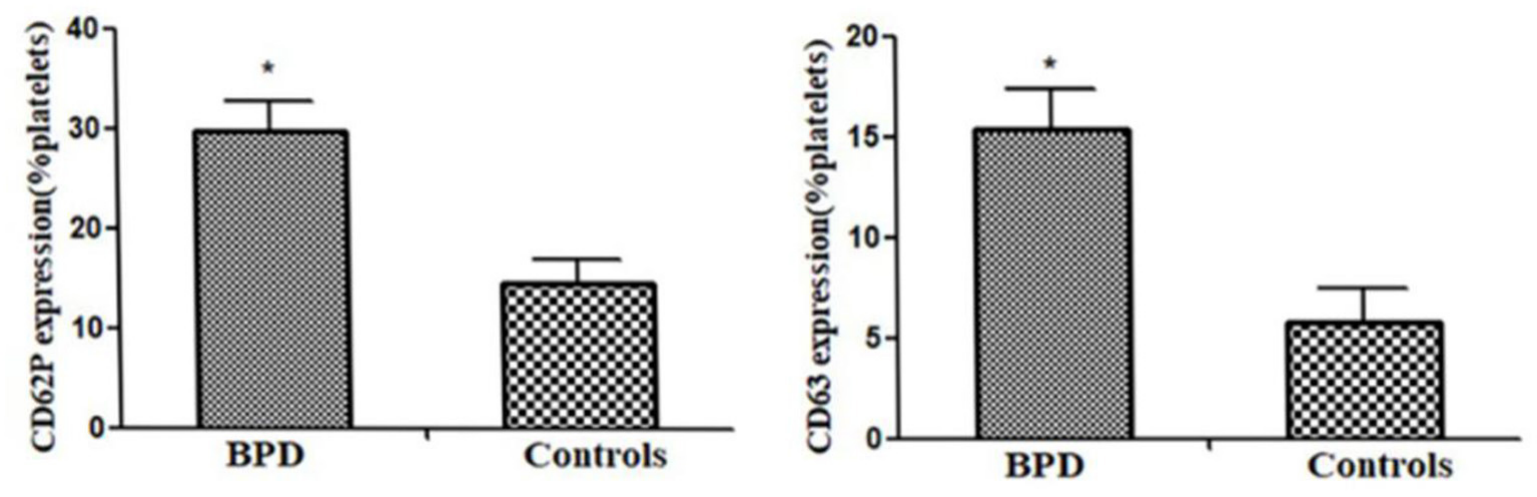
CD62P and CD63 expression in infants of BPD and control groups (all *p* < 0.05). *p < 0.05.

When compared with the controls, premature infants with BPD had significantly greater expressions of CD62P [BPD vs. controls: 29.70 (3.1%) platelets vs. 14.5 (2.5%) platelets, *p* = 0.023] and CD63 [BPD vs. controls: 15.4 (2.0%) platelets vs. 5.8 (1.7%) platelets, *p* = 0.015)], as shown in Figures 4–6.

### Plasma Thrombopoietin Concentration

**Figure 7:**
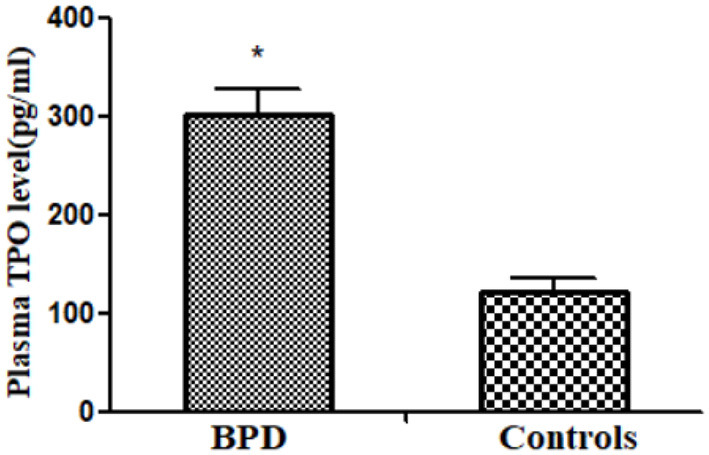
Plasma thrombopoietin (TPO) level in infants of BPD and control groups (*p* = 0.032). *p < 0.05.

Circulating MK count in the BPD group was significantly higher than that in the control group [BPD vs. controls: 301.4 (25.9) pg/ml vs. 120.4 (14.2) pg/ml, *p* = 0.032], as shown in Figure 7.

### Plasma Thrombopoietin Response to Thrombocytopenia in Infants With Bronchopulmonary Dysplasia

**Figure 8:**
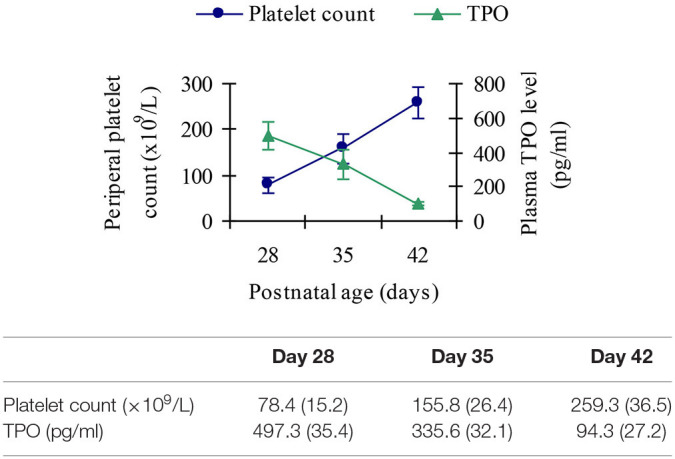
Response of plasma TPO to thrombocytopenia in infants with BPD.

TPO levels showed an inverse relationship with peripheral platelet count, peaking near the nadir and decreasing as platelet count increased, as shown in Figure 8.

## Discussion

In this prospective study, we studied the relevant indicators of platelet metabolism in premature infants with BPD and analyzed its relationship with the occurrence of BPD. Our finding in this study was consistent with our observation in the rat model (15).

In previous studies (16, 17), we have known that CD62p and CD63 are sensitive indicators of platelet activation, and we found that CD62p and CD63 were increased in premature infants who developed BPD compared with those who did not, and the platelet count in the BPD group was significantly lower than in the non-BPD group. Our observation suggested that in infants with BPD, platelet consumption was at least partly responsible for the low peripheral platelet count. The new type of BPD is characterized by alveolar and microvascular dysplasia (5), and endothelial cell injury will impair lung vascular and alveolar growth. Previous workers have reported that pulmonary endothelium was the earliest cell damaged by oxygen free radicals (18), which was followed by an influx of inflammatory cells, such as neutrophils.

Once endothelial damage occurs, the activated platelets cause inflammation by the migration of central granulocytes, which play a key role in the arrested lung development (19). In addition, the intercellular adhesion molecule-1, one of the markers in estimating neutrophil attachment to endothelial cells, was elevated in infants with BPD (20). Furthermore, the vascular endothelial growth factor, one of the markers in evaluating the growth and repair of vascular endothelial cell, was lower in infants with BPD than those without BPD (21). In addition, activated polymorphonuclear leukocytes can cause lung tissue injury by the release of toxic oxygen radicals, all of which can further damage the endothelium and increase platelet activation (22). These observations, together with ours, indicated that pulmonary endothelial damage in infants with BPD may lead to platelet activation and, most likely, increased platelet consumption, resulting in the reduction of peripheral platelet count. Therefore, inhibition of platelet activation may improve the occurrence of BPD.

Our previous study (15) confirmed that the lung is an important site for MK fragmentation and platelet release by comparing the MK and platelet counts between the pre-pulmonary and post-pulmonary blood in rats. Using state-of-the-art intravital microscopy, Lefracais et al. (23) observed the dynamic release of platelets by intravascular megakaryocytes in the lung microcirculation of mice, and intravascular lung megakaryocytes account for about 50% of total platelet production. In this study, as the central arterial and venous catheters were rarely simultaneously available in infants at their postnatal 28 days, we were not able to estimate the platelet and MK count at the pre-pulmonary and post-pulmonary circulation. Thus, we cannot confirm the effect of lung damage in BPD on platelet production. However, it is possible that in infants with BPD, there were lots of pulmonary capillary beds or the damaged endothelium was unable to retain MK for platelet release. On the other hand, mechanical ventilation may damage the pulmonary blood–gas barrier, which causes leakage of fluid, protein, and blood cells into tissue and air spaces. This leakage may result in leakage of MK cells into the alveoli and impairment of MK fragmentation into platelets due to functional impairment of the filtration and sequestration functions of pulmonary capillaries. In this experiment, we found that circulating MK count in the preterm with BPD was higher than that without BPD. The circulating MK, reflecting the MK progenitor in bone marrow, suggests that thrombocytopenia may initiate a feedback mechanism to stimulate the generation of bone marrow megakaryocytes, to increase the number of megakaryocytes in circulation.

Thrombocytopenia is the common complication of newborns in NICU. To our knowledge, TPO is a major regulator of platelet production. So far, there are no reports on plasma TPO level in infants with BPD or its relationship with platelet count. Therefore, in our study, serial TPO and platelet count measurements were obtained in some subjects. Platelet count in infants with BPD showed significantly increased plasma TPO level when compared with the infants in the control group. Our finding that TPO showed a decrease at the time of resolution of thrombocytopenia has also been observed in both adults and children (24, 25). The mechanism might be that plasma TPO concentration is dependent on circulating platelet mass (26). Elevated platelet levels lead to increased binding of cytokines to platelet receptors, which increases the activity of antiplatelet antibodies and reduces plasma concentration. Conversely, lower platelet levels lead to decreased absorption and catabolic metabolism, which leads to higher plasma TPO concentrations. This theory supports our findings.

In conclusion, platelets may play an important role in the formation and development of pulmonary microvascularization in premature infants with BPD. The mechanism may be related to platelet activation and TPO regulation. However, the specific mechanism of TPO-regulating platelets in infants with BPD needs to be further studied, which provides a new idea for the study of BPD.

## Data Availability Statement

The original contributions presented in the study are included in the article/supplementary material, further inquiries can be directed to the corresponding authors/s.

## Ethics Statement

The studies involving human participants were reviewed and approved by Guangdong Women and Children Hospital Ethics Committee. Written informed consent to participate in this study was provided by the participant's legal guardian/next of kin.

## Author Contributions

LYan and ZR conceived and coordinated the study, and wrote the paper. LYan, JW, XX, and LYang performed the experiments. FX and WG collected and analyzed the data. JM collected the references. JY edited the manuscript and provided guidance. All authors reviewed the results and approved the final version of the manuscript.

## Funding

This work was supported by the Guangdong Natural Found project (2020A1515010266) and the National Natural Science Foundation of China (81873847). The funders of the study had no role in study design, data collection or analysis, decision to publish, or preparation of the manuscript. The corresponding author had full access to all the data in the study and had final responsibility for the decision to submit it for publication.

## Conflict of Interest

The authors declare that the research was conducted in the absence of any commercial or financial relationships that could be construed as a potential conflict of interest.

## Publisher's Note

All claims expressed in this article are solely those of the authors and do not necessarily represent those of their affiliated organizations, or those of the publisher, the editors and the reviewers. Any product that may be evaluated in this article, or claim that may be made by its manufacturer, is not guaranteed or endorsed by the publisher.

## References

[1] Stoll BJ Hansen NI Bell EF Walsh MC Carlo WA Shankaran S. Trends in care practices, morbidity, and mortality of extremely preterm neonates, 1993-2012. JAMA. (2015) 314:1039–51. 10.1001/jama.2015.10244 26348753 PMC4787615

[2] Postma DS Bush A van den Berge M. Risk factors and early origins of chronic obstructive pulmonary disease. Lancet. (2015) 385:899–909. 10.1016/S0140-6736(14)60446-3 25123778

[3] Moschino L Carraro S Baraldi E. Early-life origin and prevention of chronic obstructive pulmonary diseases. Pediatr Allergy Immunol. (2020) 31:16–8. 10.1111/pai.13157 32017219

[4] Islam JY Keller RL Aschner JL Hartert TV Moore PE. Understanding the short- and long-term respiratory outcomes of prematurity and bronchopulmonary dysplasia. Am J Respir Crit Care Med. (2015) 192:134–56. 10.1164/rccm.201412-2142PP 26038806 PMC4532824

[5] Thébaud B Goss KN Laughon M Whitsett JA Abman SH Steinhorn RH. Bronchopulmonary dysplasia. Nat Rev Dis Primers. (2019) 5:78–131. 10.1038/s41572-019-0127-7 31727986 PMC6986462

[6] Jiménez J Richter J Nagatomo T Salaets T Quarck R Wagennar A. Progressive vascular functional and structural damage in a bronchopulmonary dysplasia model in preterm rabbits exposed to hyperoxia. Int J Mol Sci. (2016) 17:e1776. 10.3390/ijms17101776 27783043 PMC5085800

[7] Kroll MH Afshar-Kharghan V. Platelets in pulmonary vascular physiology and pathology. Plum Circ. (2012) 2:291–308. 10.4103/2045-8932.101398 23130099 PMC3487299

[8] Bolouki MK Zarkesh M Kamali A Dalili S Heidarzadeh A Rad AH. The association of mean platelet volume with intra ventricular hemorrhage and broncho pulmonary dysplasia in preterm infants. J Pediat Hematol Onc. (2015) 15:227–32. 26985356 PMC4779158

[9] Dani C Poggi C Barp J Berti E Fontanelli G. Mean platelet volume and risk of bronchopulmonary dysplasia and intraventricular hemorrhage in extremely preterm infants. Am J Perinatol. (2011) 28:551–7. 10.1055/s-0031-1274503 21404166

[10] Chen X Li H Qiu X Yang CZ Walther FJ. Neonatal hematological parameters and the risk of moderate-severe bronchopulmonary dysplasia in extremely premature infants. BMC Pediatr. (2019) 19:138–44. 10.1186/s12887-019-1515-6 31039810 PMC6489335

[11] Curley A Stanworth SJ Willoughby K Fustolo-Gunnink SF Venkatesh V Hudson C. Randomized trial of platelet-transfusion thresholds in neonates. N Engl J Med. (2019) 3:242–51. 10.1056/NEJMoa1807320 30387697

[12] Ehrenkranz RA Walsh MC Vohr BR Jobe AH Wright LL Fanaroff AA. Validation of the National Institutes of Health consensus definition of bronchopulmonary dysplasia[J]Pediatrics. (2005) 116:1353–60. 10.1542/peds.2005-0249 16322158

[13] Gomella T Cummingham M Fabien E. Neonatology. 6th ed. New York, NY: Lange (2009).

[14] Yang J Zhang HC Niu JM Mu XP Zhang XL Ying Liu Y. Impact of preeclampsia on megakaryocytopoesis and platelet homeostasis of preterm infants. Platelets. (2016) 27:123–7. 10.3109/09537104.2015.1048213 26083681

[15] Yang J Yang M Xu F Li K Lee SKM Ng PC. Effects of oxygen-induced lung damage on megakaryocytopoiesis and platelet homeostasis in a rat model. Pediatr Res. (2003) 54:344–52. 10.1203/01.PDR.0000079186.86219.29 12815110

[16] Ergelen M Uyarel H. Plateletcrit: a novel prognostic marker for acute coronary syndrome. Int J Cardiol. (2014) 177:161. 10.1016/j.ijcard.2014.09.054 25499366

[17] Taylor ML Misso NL Stewart GA Thompson PJ. differential expression of platelet activation markers CD62P and CD63 following stimulation with PAF, arachidonic acid and collagen. Platelets. (2009) 6:394–401. 10.3109/09537109509078478 21043771

[18] Mittal M Siddiqui MR Tran K Reddy SP Malik AB. Reactive oxygen species in inflammation and tissue injury. Antioxid Redox Signal. (2014) 20:1126–67. 10.1089/ars.2012.5149 23991888 PMC3929010

[19] Bui CB Pang MA Sehgal A Theda C Lao JC Berger PJ. Pulmonary hypertension associated with bronchopulmonary dysplasia in preterm infants. J Reprod Immunol. (2017) 124:21–9. 10.1016/j.jri.2017.09.013 29035757

[20] Wang XH Jia HL Deng L Huang WM. Astragalus polysaccharides mediated preventive effects on bronchopulmonary dysplasia in rats. Pediatr Res. (2014) 76:347–54. 10.1038/pr.2014.107 25029259

[21] Baker CD Abman SH. Impaired pulmonary vascular development in bronchopulmonary dysplasia. Neonatology. (2015) 107:344–51. 10.1159/000381129 26044102 PMC4469359

[22] Middleton EA Rondina MT Schwertz H Zimmerman GA. Amicus or adversary revisited: platelets in acute lung injury and acute respiratory distress syndrome. Am J Respir Cell Mol Biol. (2018) 59:18–35. 10.1165/rcmb.2017-0420TR 29553813 PMC6039872

[23] Lefrancais E Ortiz-Munoz G Caudrillier A Mallavia B Liu FC Sayah DM. The lung is a site of platelet biogenesis and a reservoir for haematopoietic progenitors. Nature. (2017) 544:105–9. 10.1038/nature21706 28329764 PMC5663284

[24] Temel T Cansu DU Temel HE Ozakyol AH. Serum thrombopoietin levels and its relationship with thrombocytopenia in patients with cirrhosis. Hepat Mon. (2014) 14:e18556–9. 10.5812/hepatmon.18556 24976834 PMC4071317

[25] Del Vecchio GC Giordano P Tesse R Piacente L Altomare M. De Mattia, D. Clinical significance of serum cytokine levels and thrombopoietic markers in childhood idiopathic thrombocytopenic purpura. Blood Transfus. (2012) 10:194–9. 10.2450/2011.0055-11 22153687 PMC3320780

[26] de Graaf CA Kauppi M Baldwin T Hyland CD Metcalf D Willson TA. Regulation of hematopoietic stem cells by their mature progeny. Proc Natl Acad Sci U S A. (2010) 107:21689–94. 10.1073/pnas.1016166108 21115812 PMC3003054

